# Pulmonary Tuberculosis is Associated with Elevated Risk of Lung cancer in Korea: The Nationwide Cohort Study

**DOI:** 10.7150/jca.37022

**Published:** 2020-01-20

**Authors:** Chang-Mo Oh, Yun-Ho Roh, Dohee Lim, Hyun-Joo Kong, Hyunsoon Cho, Bin Hwangbo, Young-Joo Won, Kyu-Won Jung, Kyungwon Oh

**Affiliations:** 1 Department of Preventive Medicine, School of Medicine, Kyung Hee University, Seoul, Republic of Korea.; 2 Cancer Registration and Statistic Branch, National Cancer Control Institute, National Cancer Center, Goyang, Republic of Korea.; 3 Division of Health and Nutrition Survey, Centers for Disease Control and Prevention, Cheongju, Republic of Korea.; 4 Department of Cancer Control and Population Health, National Cancer Center Graduate School of Cancer Science and Policy, National Cancer Center, Goyang, Korea.; 5 Center for lung Cancer, National Cancer Center, Goyang, Republic of Korea.

**Keywords:** Lung neoplasms, tuberculosis, smoking, incidence

## Abstract

**Objective:** Although previous studies suggest that previous pulmonary tuberculosis was associated with increased risk of lung cancer. It remains controversial whether pulmonary tuberculosis is a risk factor for lung cancer. Our study was aimed to examine the association between pulmonary tuberculosis and lung cancer risk in Korean.

**Methods:** The Korean National Health and Nutrition Examination Survey database was linked with the Korean National Cancer Incidence Database to examine the occurrence of pulmonary tuberculosis and lung cancer. The linked databases were also merged with causes of death database of Statistics Korea. The Cox-proportional hazards model was used to estimates the hazard risk of lung cancer for Korean adults aged ≥40 years with pulmonary tuberculosis.

**Results:** Of 20,252 total participants, 2,640 (13.0%) had old pulmonary tuberculosis (a medical history of pulmonary tuberculosis or radiologically inactive tuberculosis). After adjusting for all covariates, the hazard ratio of lung cancer among patients with old pulmonary tuberculosis was 3.24 (95% CI, 1.87‒5.62) compared to the control group. According to smoking status, the hazard ratios of lung cancer for never smokers, ex-smokers, and current smokers among participants with old pulmonary tuberculosis were 3.52 (95% CI, 1.17‒10.63), 2.16 (95% CI, 0.89‒5.24), and 3.71 (95% CI, 1.49‒9.22) compared to the control group, respectively.

**Conclusions:** Korean adults with old pulmonary tuberculosis have a higher risk of lung cancer, compared to general population without pulmonary tuberculosis.

## Introduction

Lung cancer is the most common cause of cancer death worldwide 1. Globally, an estimated 1.6 million people died from lung cancer in 2012 1. In South Korea, lung cancer is also the leading cause of death among cancer patients 2. Pulmonary tuberculosis is the 12^th^ leading cause of death worldwide and the 3rd most common cause of infectious disease deaths worldwide 3. In fact, the disease burden of death due to tuberculosis has surpassed that of lung cancer 3. Especially, South Korea has the highest incidence and mortality rate of pulmonary tuberculosis among OECD (Organisation for Economic Cooperation and Development) countries 4.

In recent years, epidemiological evidence concerning the association between pre-existing pulmonary tuberculosis and lung cancer has accumulated 5-12, however, this evidence is conflicting 6,13,14. Pulmonary tuberculosis is often found before or after the diagnosis of lung cancer 15, and there might be increased effort to find lung disease in patients with pulmonary tuberculosis by chest X-ray 14,16. In addition to overestimating the association between pulmonary tuberculosis and lung cancer, this relationship may be also underestimated, because many patients could have pulmonary tuberculosis without a diagnosis, specific symptoms, or awareness 17. In South Korea in particular, pulmonary tuberculosis is endemic, so many people may have lived without being aware of their previous pulmonary tuberculosis.

Therefore, our study aims to investigate the association between pulmonary tuberculosis and the risk of lung cancer in the Korean general population using the Korea National Cancer Incidence Database (KNCIDB), which was linked to the Korean National Health and Nutritional Examination Survey (KNHANES) database and cause of death data from Statistics Korea.

## Methods

### Data sources

In order to ascertain the incidence of lung cancer and pulmonary tuberculosis, a retrospective cohort study was conducted by linking two large national databases - the KNHANES database was merged with KNCIDB from the Korea Central Cancer Registry. The KNHANES is a nationwide representative cross-sectional survey that has been conducted annually since 2007 by the Korean Center for Disease Control and Prevention 18,19. The contents of KNHANES are divided into three parts: health interviews, health examinations, and nutrition surveys. In this study, we used a pooled KNHANES dataset from 2008 to 2013. The Korea Central Cancer Registry collects national cancer incidence data 2, The completeness of KNCIDB from the Korea Central Cancer Registry was estimated to be 97.8% complete in 2014 20. Incidence data from 1993 to 2014 were obtained from the KNCIDB. Finally, the merged data was linked to the cause of death data from 2008 to 2014 from Statistics Korea, which records complete death statistics. Ethics approval for the research protocol was obtained from the institutional review board (IRB) of the National Cancer Center (IRB No: NCC2017-0241, Goyang, Republic of Korea).

### Old pulmonary tuberculosis

In our study, we considered both a past medical history of pulmonary tuberculosis diagnosed by doctor and inactive pulmonary tuberculosis based on chest X-ray as old pulmonary tuberculosis 11. Chest X-ray was conducted for people ≥15 years old in KNHANES, excluding women who were pregnant or likely to be pregnant. Chest X-ray was taken with deep breathing. Two radiology experts performed multiple readings to determine the presence of pulmonary disease. An expert radiologic assessment of inactive pulmonary tuberculosis was classified as old pulmonary tuberculosis in this study 18. A sensitivity analysis was performed for each cases of old pulmonary tuberculosis (based on past medical history or chest X-ray) to examine the robustness of the association between old pulmonary tuberculosis and lung cancer risk.

Lung cancer cases were defined as ICD-10 “C33” and “C34”. To define new incidence only, we excluded people from KNCIDB diagnosed with lung cancer before 2008.

### Follow-up method

From 2008 to 2013, the date of participation in the KNHANES was the date of start of follow-up for each participant, after excluding patients diagnosed with lung cancer before the date of study participation. Data were examined to determine the participants' status until December 31, 2014 using data from Statistics Korea. The main outcome was the new incidence of lung cancer from 2008 to 2014. Therefore, those who was participated in KNHANES and died between 2008 and 2014 were censored. Participants who were not diagnosed with lung cancer or who did not die were followed up until December 31, 2014.

### Study participants

Participants ≥40 years old were included, because there were rare lung cancer cases among people <40 years old. We excluded people who were diagnosed with lung cancer before participating the KNHANES survey (N = 136), people who lacked information about education, income level, smoking status, body mass index (BMI), and physical activity (N = 1,379). The missing number and percentage of smoking, BMI, income, education was as follows: smoking status (missing n: 901), BMI (missing n: 92), education (missing n: 880), income level (missing n: 436), physical activity (missing n: 905). People who lacked questionnaire responses regarding past history of pulmonary tuberculosis (N = 3,965) were also excluded from final study participants. Those diagnosed with lung cancer within 6 months after KNHANES survey were excluded (N = 11), because these patients were likely to be affected by factors other than pulmonary tuberculosis. Finally, 20,252 participants were included in the final analysis (Supplementary figure 1).

### Measurement or categorization of variables

Information about past medical history of pulmonary tuberculosis, smoking history, alcohol intake, and physical activity was obtained from KHANES 18. Past medical history of pulmonary tuberculosis was defined as having been diagnosed with pulmonary tuberculosis from a doctor. Smoking status was divided into never smoker, ex-smoker, and current smoker. Moderate or vigorous physical activity was defined as performing moderate-intensity physical activity for more than 30 minutes/day 5 times/week or vigorous-intensity physical activity for more than 20 minutes/day 3 times/week 21,22. Equivalent household income was calculated as the square root of the monthly household income divided by the square root of number of family members in the household 22. Four income quartile groups were established. Education level was categorized as middle school or lower, high school, and college or higher. BMI was calculated as weight (kg) divided by square of height (m).

### Statistical analysis

All analyses were conducted using sampling weights considering the complex survey design. The baseline characteristics of the study participants are presented as means (standard error) or numbers (percentage) by old pulmonary tuberculosis. The independent t-test and the chi-square test were used to test the difference in continuous variables and categorical variables, respectively, between patients with old pulmonary tuberculosis and the control group. Then, we compared the number and percentage of lung cancer cases between patients with old pulmonary tuberculosis and the control group who did not have pulmonary tuberculosis by age group (<60, ≥60 years old), sex, and smoking history. The cumulative incidence risk of lung cancer was calculated as incidence cases per 100,000 person-years, and was assessed between patients with old pulmonary tuberculosis and the control group by an incidence rate ratio. Kaplan-Meier plot was used and log-rank test was examined the difference between two groups. Cox proportional hazards models were applied to estimate hazard ratios (HRs) and 95% confidence intervals (CIs) for the associations between patients with old pulmonary tuberculosis and an elevated risk of lung cancer. The first model included pulmonary tuberculosis variables only as the independent variable. In the second model, sex and age were adjusted. In the third model, age, sex, education level, income level, smoking status, BMI, and physical activity were adjusted as covariates. Subgroup analysis was performed to examine the association between pulmonary tuberculosis and histological types of lung cancer. Histological types were divided into squamous cell carcinoma, adenocarcinoma and others by WHO/IARC classification of tumors 23.

P-values <0.05 were considered statistically significant. All statistical analysis was conducted using SAS 9.4 (SAS Institute, Cary, NC, U.S.A.) and STATA version 14 (StataCorp LP, College Station, TX, USA).

## Results

### Baseline characteristics

The general characteristic of study participants were presented in table 1. Of the 20,252 total participants, 2,640 (13.0%) had old pulmonary tuberculosis (men: 1,519, women: 1,121). Compared to the general population without pulmonary tuberculosis, patients with old pulmonary tuberculosis were older (62.92 years), had a lower BMI (22.89 kg/m^2^) (p<0.001, p<0.001, respectively). Patients with old pulmonary tuberculosis had more men and current smokers compared to the general population without pulmonary tuberculosis (p<0.001, p<0.001, respectively). Patients with old pulmonary tuberculosis had a lower education level and income level compared to the general population without pulmonary tuberculosis (p<0.001, p<0.001, respectively). There was no significant difference in physical activity level between patients with old pulmonary tuberculosis and the general population without pulmonary tuberculosis (p = 0.059).

### Lung cancer risk for patients with old pulmonary tuberculosis

**Figure 1:**
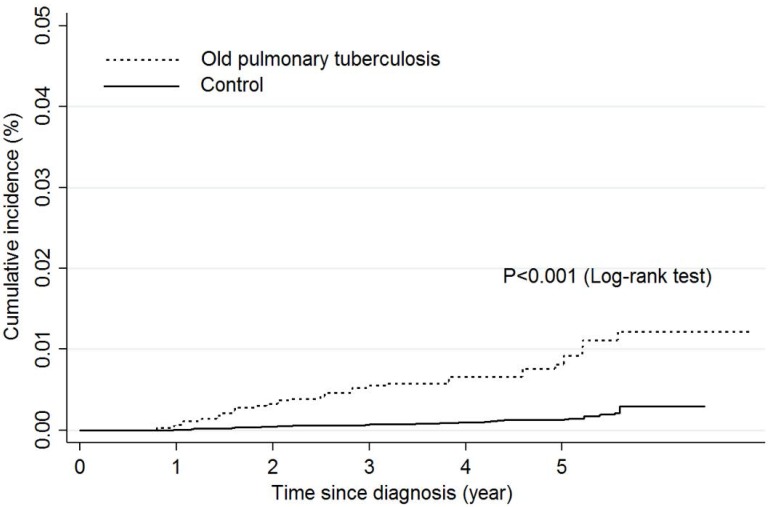
Comparison of incidence rate of lung cancer between patients with old pulmonary tuberculosis and control group

Sixty-five patients (0.3%) were newly diagnosed with lung cancer until December 31, 2014 (Table 2). Of these 65 patients, 27 (1.0%) had pulmonary tuberculosis and 38 (0.2%) were in the control group without pulmonary tuberculosis. The overall relative risk of lung cancer for patients with old pulmonary tuberculosis was 5.66 (95% CI: 3.17‒10.12) compared to the control group (Table 2, Figure 1). According to sex, the relative risks of lung cancer in men and women with old pulmonary tuberculosis were 5.28 (95% CI: 2.65‒10.50) and 4.06 (95% CI: 1.19‒13.89) compared to the control group. By age group, the relative risk of lung cancer among adults with old pulmonary tuberculosis under 60 years old was 2.19 (95% CI: 0.53‒9.12), but this was not statistically significant. The relative risk of lung cancer in adults with old pulmonary tuberculosis over 60 years old was 5.51 (95% CI: 3.03‒10.01) compared to the control groups. Regarding smoking status, the relative risks of lung cancer for never smokers, ex-smokers, and current smokers with old pulmonary tuberculosis were 4.82 (95% CI: 1.60‒14.56), 3.56 (95% CI: 1.13‒11.16) and 7.10 (95% CI: 2.94‒17.13) compared to the control group, respectively. By histological type of lung cancer, the relative risk of people with old pulmonary tuberculosis for squamous cell carcinoma, adenocarcinoma and other types of lung cancer were 3.39 (95% CI: 0.95‒12.05), 4.32 (95% CI: 1.77‒10.58) and 13.07 (95% CI: 4.96‒34.50). The incidence rate of lung cancer was 31.2 per 100,000 person-years in the control group, compared to 177.6 per 100,000 person-years in patients with old pulmonary tuberculosis.

### Hazard ratio of lung cancer for patients with old pulmonary tuberculosis

The HRs and 95% CIs of lung cancer were calculated after adjusting for multiple covariates (Table 3). After adjusting for age and sex, the HR of lung cancer for patients with old pulmonary tuberculosis was 3.31 (95% CI: 1.89‒5.78), and after adjusting for age, sex, smoking status, BMI, physical activity, education level, and income level, the HR was 3.24 (95% CI: 1.87‒5.62) compared to controls.

### Subgroup analysis by smoking status

We performed a subgroup analysis after stratifying subjects by smoking status to assess whether the relationship between old pulmonary tuberculosis and risk of lung cancer varied by smoking status (Table 4). For never smokers and current smokers, the adjusted HRs of lung cancer for patients with old pulmonary tuberculosis were 3.52 (95% CI: 1.17‒10.63) and 3.71 (95% CI: 1.49‒9.22) compared to controls. However, the adjusted HRs for ex-smokers with old pulmonary tuberculosis was 2.16 (95% CI: 0.89‒5.24) compared to controls.

### Subgroup analysis by histological types of lung cancer

We performed a subgroup analysis by histological types of lung cancer (Supplementary table 1). For squamous cell carcinoma, the adjusted HRs of patients with old pulmonary tuberculosis were 2.05 (95% CI: 0.64‒6.60), whereas the adjusted HRs of patients with old pulmonary tuberculosis for adenocarcinoma was 3.18 (95% CI: 1.35‒7.51) compared to people without old pulmonary tuberculosis. Other types of lung cancer (including small cell carcinoma, sarcoma, etc) showed the strong association with old pulmonary tuberculosis (Adjusted HR: 4.54 (95% CI: 1.61‒12.81).

### Sensitivity analysis for past medical history of pulmonary tuberculosis and pulmonary tuberculosis detected by chest X-ray

A sensitivity analysis was performed to determine the robustness of the relationship between pulmonary tuberculosis and risk of lung cancer according to different types of pulmonary tuberculosis: past medical history of pulmonary tuberculosis defined by questionnaire only, and pulmonary tuberculosis detected by chest X-ray only (Table 5). After adjusting for all covariates, the adjusted HR of lung cancer risk for patients with past medical history of pulmonary tuberculosis defined by questionnaire only was 1.71 (95% CI: 0.86‒3.39) and for pulmonary tuberculosis detected by chest X-ray only was 2.86 (95% CI: 1.55‒5.27) compared to controls.

## Discussion

In this study, the incidence density of lung cancer in patients with old pulmonary tuberculosis was 177.6 per 100,000 person-years, much higher than that of 31.2 per 100,000 person-years among people without pulmonary tuberculosis. In addition, the HR of lung cancer for patients with old pulmonary tuberculosis was 3.24 compared to the control group, after adjusting for possible covariates.

Although pulmonary tuberculosis seems to be closely associated with lung cancer, their causal relationship has remained controversial 5,14. One major mechanism is that chronic tuberculosis infections lead to scarring and recurrent chronic inflammation, which leads to lung metaplasia and cancer 24,25. From this point of view, our study finding, which showed stronger association with lung cancer among patients diagnosed by chest X-ray than among patients defined by past medical history, seems to support “scar cancer” theory.

However, observational studies could not guarantee a causal relationship. For example, surveillance bias, classification bias, or confounding factors such as smoking may have impacted the results of previous studies 14. In addition, previous epidemiological studies did not have consistent conclusions 6,10,14. Therefore, it remains controversial whether pulmonary tuberculosis increases the risk of lung cancer. To overcome these problems, our study conducted a stratification analysis by smoking status, which is a well-known major risk factor of lung cancer. As a result, most of our study findings, with the exception of the results for ex-smokers, consistently showed that patients with pulmonary tuberculosis had an increased risk of lung cancer.

Our study also showed that the risk of lung cancer for never-smokers with old pulmonary tuberculosis was similar to that of smokers, which is consistent with findings of previous studies. Recent meta-analysis showed that the point estimate for lung cancer risk among never-smokers with pulmonary tuberculosis was similar to the overall risk 5,6. These findings suggest that pulmonary tuberculosis could be a risk factor for lung cancer independent of smoking status.

In our study finding, old pulmonary tuberculosis was more strongly associated with adenocarcinoma than squamous cell carcinoma. Previous meta-analysis for the 41 observational studies also showed the significant association between prior tuberculosis exposure and adenocarcinoma of lung 5. In addition, recent studies report that higher expression of EGFR mutations in patients with pulmonary tuberculosis, and EGFR mutations have been commonly observed in never smokers, in East Asians and in adenocarcinoma of lung 26, 27.

However, it was difficult to explain why other types of lung cancer, including small cell carcinoma and sarcoma, were strongly associated with pulmonary tuberculosis in our study. It may be affected by the chance due to small number of subtypes of lung cancer or misclassification bias.

### Strengths and limitations

One of major strengths of our study is that we adjusted for socioeconomic status, which can cause bias in the relationship between pulmonary tuberculosis and lung cancer. The prevalence of tuberculosis is known to be closely related to poverty 28, but this relationship has been ignored in previous association studies. In our study, the prevalence of pulmonary tuberculosis was increased at lower income levels. However, interestingly, the risk of lung cancer was decreased at lower income levels, which is in contrast to some studies showing that lower socioeconomic status was associated with an elevated risk of lung cancer 29,30. Another strength of our study is that the study participants are representative of all Korean adults, so our findings can be generalized to the entire Korean population. Few studies of the association between pulmonary tuberculosis and the risk of lung cancer are representative of an entire population. We also examined the association between old pulmonary tuberculosis and lung cancer using both a past medical history of pulmonary tuberculosis and inactive pulmonary tuberculosis based on chest X-ray, and we found consistent results in the direction of old pulmonary tuberculosis and the risk of lung cancer. In addition, we could show the association between old pulmonary tuberculosis and lung cancer by histological types of lung cancer.

Although our study was a representative nationwide cohort study, it has some limitations. First, there was relatively small number of lung cancer patients due to the short follow-up period. Therefore, we could not investigate the association between old pulmonary tuberculosis and subtype of lung cancer (small cell lung cancer, squamous cell carcinoma, adenocarcinoma). Second, there may be misclassification of pulmonary tuberculosis, although we used both the questionnaire for past medical history and chest X-ray to define pulmonary tuberculosis. In addition, there was no sputum examination and no validity test of how well past history or chest X-ray matched to the medical record. Especially, the detection of pulmonary tuberculosis only by chest X-ray included inactive pulmonary tuberculosis, so there was a possibility of overestimating the prevalence of pulmonary tuberculosis. Third, the scar of pulmonary tuberculosis detected by chest X-ray may hide early cancer lesions. Co-existing pulmonary tuberculosis was frequently found among patients diagnosed with lung cancer 15. In addition, our study follow-up period was relatively short (mean follow-up period 3.9 years).

## Conclusion

Our research is meaningful because our study implies that pulmonary tuberculosis could be an independent risk factor for lung cancer in the general Korean population. These results are particularly meaningful in South Korea, which has the highest incidence and mortality of pulmonary tuberculosis among OECD countries. Our study findings suggest that the prevention and management of pulmonary tuberculosis is also important for the future prevention of lung cancer. Further efforts are necessary to prevent and screen lung cancer in patients with pulmonary tuberculosis in South Korea.

## Supplementary Material

Supplementary figures and tables.

## Figures and Tables

**Table 1:** Baseline characteristics of patients with old pulmonary tuberculosis

Characteristics	Old Tb patients^*^(N=2,640)		Control (N=17,612)		*p*-value^†^
	Mean	(SE)	Mean	(SE)	
**Follow-up years(year)**	3.85	(0.05)	4.00	(0.04)	<.0001
**Age(year)**	62.92	(0.30)	57.77	(0.14)	<.0001
**BMI(kg/m^2^)**	22.89	(0.07)	24.15	(0.03)	<.0001
	**No**	**(%)**	**No**	**(%)**	
**Sex**					
Men	1,519	(59.73)	7,193	(46.70)	<.0001
Women	1,121	(40.27)	10,419	(53.30)	
**Smoking status**					
Never smoker	1,274	(45.79)	11,045	(57.70)	<.0001
Ex-smoker	814	(29.90)	3,410	(20.12)	
Current smoker	552	(24.32)	3,157	(22.17)	
**Education**					
Middle school or lower	1,534	(52.69)	9,173	(45.62)	<.0001
High school	672	(28.91)	5,117	(32.91)	
College or higher	434	(18.40)	3,322	(21.47)	
**Income level**					
1st quartile(highest)	510	(22.65)	4,666	(28.54)	<.0001
2nd quartile	641	(26.47)	4,501	(28.07)	
3rd quartile	688	(26.22)	4,342	(24.87)	
4rd quartile(lowest)	801	(24.66)	4,103	(18.52)	
**Moderate or vigorous physical activity**					
No	2,150	(80.33)	13,796	(78.34)	0.059
Yes	490	(19.67)	3,816	(21.66)	

Tb=Tuberculosis, BMI= Body mass index, SE=standard error. ^*^Old pulmonary tuberculosis included both a past medical history of pulmonary tuberculosis or non-active pulmonary tuberculosis based on chest X-ray. ^†^The independent t-test and the chi-square test were used to test the difference in continuous variables and categorical variables, respectively.

**Table 2:** Lung cancer risk of patients with old pulmonary tuberculosis by characteristics

Characteristics	Old Tb patients^*^			Control			IRR (95% CI)		
	Lung cancer no.	(%)	Incidence rates per 100,000 person-years	Lung cancer no.	(%)	Incidence rates per 100,000 person-years			
**Total**	27	1.0	177.6	38	0.2	31.2	5.66 (3.17-10.12)		
**Sex**									
Men	23	85.19	247.8	27	71.1	46.5	5.28 (2.65-10.50)		
Women	4	14.81	71.5	11	28.9	17.8	4.06 (1.19-13.89)		
**Age group**									
<60	3	11.11	42.3	9	23.68	17.9	2.19 (0.53-9.12)		
≥60	24	88.89	341.7	29	76.32	63.0	5.51 (3.03-10.01)		
**Smoking status**									
Never smoker	6	22.22	84.6	12	31.58	17.8	4.82 (1.60-14.56)		
Ex-smoker	10	37.04	197.8	12	31.58	54.1	3.56 (1.13-11.16)		
Current smoker	11	40.74	321.7	14	36.84	45.5	7.10 (2.94-17.13)		
**Histologic type**									
Squamous cell carcinoma	6	22.22	35.59	11	28.95	10.14	3.39 (0.95-12.05)		
Adenocarcinoma	9	33.33	65.33	19	50.00	15.24	4.32 (1.77-10.58)		
Others	12	44.44	76.72	8	21.05	5.87	13.07 (4.96-34.50)		

Tb=Tuberculosis, BMI= Body mass index, CI= Confidence interval, IRR= Incidence rate ratio. ^*^Old pulmonary tuberculosis included both a past medical history of pulmonary tuberculosis or non-active pulmonary tuberculosis based on chest X-ray.

**Table 3:** Hazard ratios and 95% CI of lung cancer risk for patients with old pulmonary tuberculosis

	Unadjusted Model	Age and sex adjusted model	Multivariable adjusted model*
	HR (95%CI)	HR (95%CI)	HR (95%CI)
**Old pulmonary tuberculosis^†^**			
No	1.00 (Reference)	1.00 (Reference)	1.00 (Reference)
Yes	5.66 (3.17-10.12)	3.31 (1.89-5.78)	3.24 (1.87-5.62)
**Sex**			
Men		1.00 (Reference)	1.00 (Reference)
Women		0.29 (0.15-0.57)	0.40 (0.20-0.77)
**Age**		1.08 (1.06-1.10)	1.08 (1.05-1.10)
**Education**			
≥colleage			1.00 (Reference)
High school			0.92 (0.24-3.48)
≤Middle school			1.12 (0.35-3.53)
**Income level**			
4st quartile(highest)			1.00 (Reference)
3st quartile			0.98 (0.28-3.44)
2st quartile			1.04 (0.37-2.89)
1st quartile(lowest)			1.07 (0.41-2.78)
**Smoking status**			
Never smoker			1.00 (Reference)
Ex-smoker			1.30 (0.59-2.85)
Current			2.27 (1.23-4.17)
**BMI(kg/m^2^)**			0.98 (0.90-1.08)
**Moderate or vigorous physical activity**			
No			1.00 (Reference)
Yes			0.65 (0.25-1.66)

HR= Hazard ratio, BMI= Body mass index, CI= Confidence interval. ^*^Adjusted for age, sex, education, income level, smoking status, bmi, moderate or vigorous physical activity. **^†^**Old pulmonary tuberculosis included both a past medical history of pulmonary tuberculosis or non-active pulmonary tuberculosis based on chest X-ray.

**Table 4:** Subgroup analysis for lung cancer risk according to smoking history

	Unadjusted Model	Age and sex adjusted model	Multivariable adjusted model*
	**HR (95%CI)**	**HR (95%CI)**	**HR (95%CI)**
**Never smoker**			
Old pulmonary tuberculosis^†^			
No	1.00 (Reference)	1.00 (Reference)	1.00 (Reference)
Yes	4.82 (1.60-14.56)	3.91 (1.40-10.93)	3.52 (1.17-10.63)
**Ex-smoker**			
Old pulmonary tuberculosis^†^			
No	1.00 (Reference)	1.00 (Reference)	1.00 (Reference)
Yes	3.56 (1.13-11.16)	2.33 (0.86-6.31)	2.16 (0.89-5.24)
**Current smoker**			
Old pulmonary tuberculosis^†^			
No	1.00 (Reference)	1.00 (Reference)	1.00 (Reference)
Yes	7.10 (2.94-17.13)	3.83 (1.59-9.26)	3.71 (1.49-9.22)

HR= Hazard ratio, BMI= Body mass index, CI= Confidence interval. ^*^Adjusted for age, sex, education, income level, smoking status, bmi, moderate or vigorous physical activity. ^†^Old pulmonary tuberculosis included both a past medical history of pulmonary tuberculosis or non-active pulmonary tuberculosis based on chest X-ray.

**Table 5:** Subgroup analysis for lung cancer risk according to according to different types of pulmonary tuberculosis

	Unadjusted Model	Age and sex adjusted model	Multivariable adjusted model†
	HR (95%CI)	HR (95%CI)	HR (95%CI)
**Past history of pulmonary tuberculosis diagnosed by doctor**			
No	1.00 (Reference)	1.00 (Reference)	1.00 (Reference)
Yes	2.84 (1.41-5.71)	1.81 (0.90-3.67)	1.71 (0.86-3.39)
**Non-active pulmonary tuberculosis on the chest X-ray**			
No	1.00 (Reference)	1.00 (Reference)	1.00 (Reference)
Yes	5.50 (2.99-10.12)	2.99 (1.64-5.47)	2.86 (1.55-5.27)

HR= hazard ratio, BMI= body mass index, CI= confidence interval. ^*^ Subgroup analysis were performed after stratifying subjects by type of pulmonary tuberculosis: a past medical history of pulmonary tuberculosis or non-active pulmonary tuberculosis based on chest X-ray. ^†^Adjusted for age, sex, education, income level, smoking status, bmi, moderate or vigorous physical activity.

## Abbreviations

OECD: Organisation for Economic Cooperation and Development
KNCIDB: The Korea National Cancer Incidence Database
KNHANES: The Korean National Health and Nutritional Examination Survey
BMI: body mass index
HR: hazard ratio
CI: confidence interval
