# Supplementary material for: Pulmonary Tuberculosis is Associated with Elevated Risk of Lung cancer in Korea: The Nationwide Cohort Study
Source: J Cancer. 2020 Jan 20;11(7):1899–906. doi: 10.7150/jca.37022 (PMC7052874; doi:10.7150/jca.37022)
Supplement: Supplementary file 1 — Supplementary figures and tables. [file jcav11p1899s1.pdf]

Supplementary table 1. Subgroup analysis for lung cancer risk by histologic type

	Squamous cell carcinoma	Adenocarcinoma	Others
	**HR (95%CI)**	**HR (95%CI)**	**HR (95%CI)**
**Old pulmonary tuberculosis†**			
No	1.00 (Reference)	1.00 (Reference)	1.00 (Reference)
Yes	2.05 (0.64-6.60)	3.18 (1.35-7.51)	4.54 (1.61-12.81)

HR= hazard ratio, CI= confidence interval

*Subgroup analysis were performed for risk of lung cancer by histologic type: squamous cell carcinoma, adenocarcinoma and others.

†Adjusted for age, sex, education, income level, smoking status, bmi, moderate or vigorous physical activity

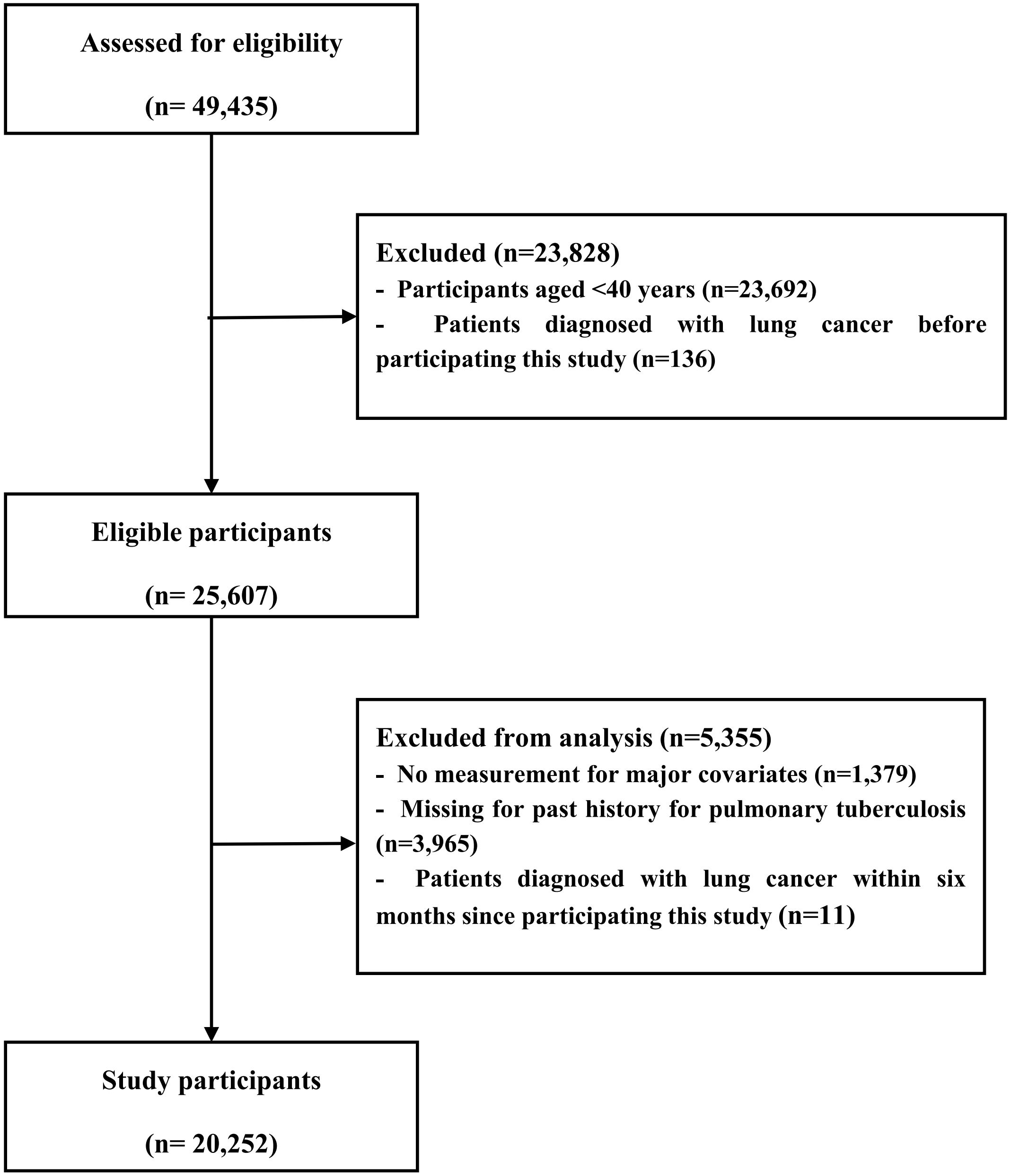

Supplementary figure 1. Flow chart for selection of study partcipants
